# Human–Environment Interactions Shape Mosquito Seasonal Population Dynamics

**DOI:** 10.3390/insects15070527

**Published:** 2024-07-12

**Authors:** Laura Blanco-Sierra, Jesús Bellver-Arnau, Santi Escartin, Simone Mariani, Frederic Bartumeus

**Affiliations:** 1 Centre d’Estudis Avançats de Blanes (CEAB-CSIC), Carrer d’Accés a la Cala St. Francesc 14, 17300 Blanes, Girona, Spain; jesus.bellver@ceab.csic.es (J.B.-A.); santi.escartin@ceab.csic.es (S.E.); mariani@ceab.csic.es (S.M.); fbartu@ceab.csic.es (F.B.); 2 Centre de Recerca Ecològica i Aplicacions Forestals (CREAF), Cerdanyola del Vallès, 08193 Barcelona, Spain; 3 Institució Catalana de Recerca i Estudis Avançats (ICREA), Passeig de Lluís Companys 23, 08010 Barcelona, Spain

**Keywords:** *Aedes*, management, mosquito, population dynamics, seasonality, vector ecology, vector control

## Abstract

**Simple Summary:**

This study investigates the factors influencing local populations of *Aedes albopictus*, a highly invasive mosquito species known for its role in spreading vector-borne diseases. Aimed at enhancing vector control effectiveness, the research focused on understanding the interplay between meteorological elements, human activity, and pest management efforts in determining mosquito abundance at local scales. Conducted over a study season at a botanical garden, the investigation utilized weekly BG-Trap data to observe adult *Ae. albopictus* dynamics. Results indicated a significant seasonal trend affected by temperature and modulated by rainfall events responsible for the observed bimodal abundance pattern. Additionally, nearby stagnant water and human presence were positively linked to mosquito abundance, while larvicide effectiveness varied with the preceding rainfall levels and application timing. Given the potential for global warming to shift weather patterns and increase the frequency of extreme events, these findings underscore the importance of integrating fine-scale ecological parameters with broader climatic trends in mosquito abatement strategies. This comprehensive approach could enhance the efficacy of interventions and mitigate the impact of mosquito-borne illnesses.

**Abstract:**

Mosquito species, including the Asian tiger mosquito, can transmit disease-causing pathogens such as dengue, Zika, and chikungunya, with their population dynamics influenced by a variety of factors including climate shifts, human activity, and local environmental conditions. Understanding these dynamics is vital for effective control measures. Our study, conducted in Jardí Botanic Marimurtra from May to November 2021, monitored *Ae. albopictus* activity using BG-Traps and investigated larval control effects. We employed Generalized Linear Mixed Models to analyze variables like weather, human presence, and larvicidal control on adult mosquito abundance. Adults of *Ae. albopictus* exhibited a seasonal pattern influenced by temperature but with bimodal peaks linked to cumulative rainfall. Proximity to stagnant water and visitor influx directly affected mosquito captures. Additionally, the effectiveness of larvicide treatments depended on interactions between preceding rainfall levels and treatment timing. Our research emphasizes the significance of studying vector ecology at local scales to enhance the efficacy of control programs and address the escalating burden of vector-borne diseases. Considering the impacts of extreme weather events and climate shifts is essential for the development of robust vector control strategies. Furthermore, our distinct findings serve as a prime illustration of utilizing statistical modeling to gain mechanistic insights into ecological patterns and processes.

## 1. Introduction

The dynamics of mosquito populations are influenced by a combination of biotic and abiotic factors, which vary in their significance across different ecological scales [1]. At the large scale, the evolving dynamics of human mobility, urban expansion, travel patterns, and climate conditions have brought about novel pathways for the transmission of disease-causing pathogens transmitted by mosquitoes, as well as variations in mosquito population trends [2,3,4,5,6]. Global warming is associated with an increasing risk of extreme weather events, recognized to pose special health hazards, including the threat of vector-borne infectious diseases. Stagnant water left over from rainfall events leads to breeding pools and an increased abundance of mosquito species. Climate-change expectations are that the frequency and intensity of rainfall events will change towards the extremes (droughts and heavy raining), altering the already complex relationship between the water cycle and the availability of mosquito developmental sites in urban areas [7,8,9].

One such example is the Asian tiger mosquito *Aedes albopictus*, native to Southeast Asia but now found across nearly all continents [10] and a vector of pathogens like dengue virus, chikungunya virus and Zika virus. This species has demonstrated remarkable adaptability by transitioning from its original habitats in tropical and subtropical forests to urban and periurban environments worldwide, establishing itself as a significant urban vector [11,12]. Key to this adaptation is biological changes, such as the shift from primarily feeding on animals (zoophily) to primarily feeding on humans (anthropophily) and the ability to breed in artificial containers (e.g., water drainers, fountains, flower pots, and tires) commonly found in urban settings [3]. Urban settings also foster optimal conditions for accelerated larval development rates, prolonged adult survival times [13], and heightened pathogen transmission risks, owing to the dense aggregation of populations. Additionally, temperate strains of *Ae. albopictus* have developed a diapause mechanism, enabling them to survive and spread into regions with cooler climates [14].

While the primary determinants for the presence of *Ae. albopictus* at continental scales have been extensively studied, there remains a lack of comprehensive understanding regarding how proximate factors modulate its seasonal variation [15]. At local scales, mosquito populations exhibit erratic and opportunistic behavior, being highly sensitive to factors such as local temperature [15], precipitation [16], the availability of developmental sites (natural or artificial) [17], and land cover [18]. Consequently, it is unclear how these components interact to shape mosquito seasonal patterns and what role different human activities play in either fostering or reducing mosquito population abundance. Mosquito life cycles respond fast to short-term weather changes like heavy rains, floods or rapid temperature shifts [19]. Notably, these local drivers, especially short-term atmospheric conditions, directly tie into the effects that climate change (i.e., changes in long-term weather patterns) can have on mosquito lifespan and behavior at broader scales. Because of all the above, to effectively implement control strategies, it is crucial to understand the ecological response of mosquito populations at the local scales. That means understanding the potential relationships between the local environmental and human-related factors, including specific control measures at the local level. Some studies [20,21,22,23,24] have already developed seasonal models at a limited scale, as they can provide valuable information in risk assessment protocols. For instance, in Camargo et al. [20], it was found that variables such as precipitation and high humidity positively affected the abundance of *Ae. albopictus*, whereas temperature and wind speed showed a negative correlation. Other studies, such as Manica et al. [24], have demonstrated that higher abundances of *Ae. albopictus* were observed in areas with greater anthropization, and that temperature and precipitation significantly influenced density fluctuations. However, none have analyzed in detail the effect of proximate environmental drivers, including human activities. Based on an intensive mosquito sampling program in a botanical garden, we untangle the determinants of the local seasonal patterns of *Ae. albopictus*.

Here, we examine key local factors impacting mosquito abundance, including weather conditions, breeding site proliferation (due to rain and human water management), the presence of humans in the area, and the impact of vector control actions with larvicide treatments on subsequent adult emergence. This comprehensive analysis can help in bringing tools to predict mosquito density and ultimately improve the effectiveness of control interventions.

## 2. Materials and Methods

### 2.1. Sampling Site

**Figure 1:**
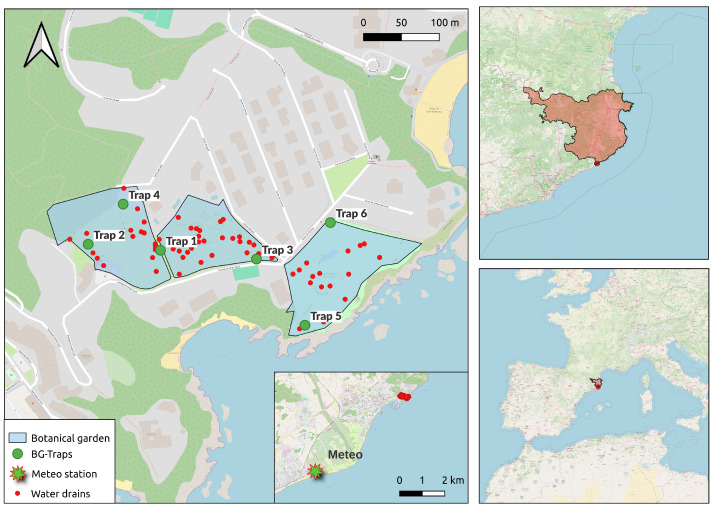
Map of the study site (province of Girona in red) and location of BG traps, water drains (N = 65) and meteorological station.

The study was conducted at the Jardí Botanic Marimurtra, a botanical garden located at the city of Blanes, Catalonia, in Northeastern Spain. This garden consists of 4 planted hectares open to visitors. The visitors-accessible space is divided into three distinct gardens, showcasing a diverse array of approximately 4000 plant species from the five continents. Consequently, each garden presents different conditions, including variations in plant composition, watering needs, wind exposure, and orientation. This diversity creates a range of environments that have the potential to influence the prevalence of mosquitoes to varying extents in each area (Figure 1).

### 2.2. Mosquito Sampling

Adult mosquitoes were collected using 6 BG-Traps (Biogents, Regensburg, Germany) with BG-lure attractant. The BG-Traps were located within the botanical garden, exposed to the different environments in the three gardens throughout the period spanning from May to November 2021 (Figure 1). We monitored the BG-Traps on a weekly basis, and sampled mosquitoes were transferred in their capture bags to the laboratory and frozen at −20 °C. We counted and identified the species and sex of the insects in the laboratory with a stereo-microscope. Among the different insect species identified, several mosquitoes were captured, including *Aedes albopictus*, *Culex pipiens*, and *Culiseta longiareolata*. However, we solely focused on *Ae. albopictus*, as it was our target species. Each trap remained connected to a continuous electricity supply throughout the entire sampling period, ensuring that captures were conducted 24 h per day.

### 2.3. Biological Control

Control measures in the botanical garden primarily involved the use of Vectomax^®^ FG (Valent Biosciences LLC, Libertyville, IL, USA), a biological treatment comprising *Bacillus thuringiensis* var. *israelensis* and *Lysinibacillus sphaericus*. This combination yields a more effective product compared to the use of *Bti* alone. It is characterized by its rapid action, sustained efficacy against mosquito larvae, and heightened toxicity attributed to the synergistic interactions between toxins, thereby preventing the development of resistances [25]. This treatment effectively targeted mosquito larvae in various aquatic habitats, including breeding sites, being relatively safe for other non-target species [26]. While the manufacturer recommended a dosage of 10 g/50 L for drainage systems and channels, we doubled the dosage during treatments to account for potential water leaching and potential product dilution caused by rainfall or watering.

The treatments were deployed 4 times at eight-week intervals, covering from 12 April to 27 September 2021. At each inspection, treatments were applied to both active and inactive breeding sites (i.e., water drains) within the botanical garden (Figure 1). Among the inactive sites, those in which no water or larvae were observed at the current inspection times but were observed in previous inspections were also treated as a precautionary measure. The monitoring of breeding sites also included plants that can accumulate water, such as bromeliads and mosses, where the presence of mosquito eggs and larvae had been previously observed. To analyze the effect of the larvicidal treatments on mosquito abundance over time, we computed the variable “weeks since treatments”, representing the number of weeks from one larvicidal treatment to the next. Notably, the days of control interventions did not coincide with mosquito collection days. Therefore, to assess the impact of the treatment interventions on mosquito abundance, we aggregated weekly count data from the traps, using the intervention week as the reference week.

In addition to Vectomax^®^ FG biological treatments, workers at the botanical garden implemented mechanical control methods periodically over the season, i.e., the selective removal of bromeliad plants in specific areas, cleaning or modifying problematic drains, and with the introduction of fish (*Gambusia affinis*) into isolated ornamental ponds within the garden.

### 2.4. Statistical Analysis

Data on minimum, average, and maximum daily temperatures (°C), relative humidity (%) and daily accumulated rainfall (mm) were obtained from the meteorological station closest to the study area. For each temporal window, we derived the means of temperature and relative humidity and the sum of the cumulative precipitation. As temperature, relative humidity, and rainfall influence not only the activity of adults at the time of trap exposure but also prior events such as hatching and larval development, we additionally computed weekly meteorological variables for the 3-week windows preceding the mosquito capture times. Furthermore, we calculated the Growing Degree Days (GDDs) (i.e., a measure of heat accumulation used to predict plant and animal development rates, such as the date on which an insect will emerge from dormancy) for both the week of sampling and for the three weeks prior to sampling times, with Tbase set at 11 °C [24,27]. To obtain the GDD variable, we used the pollen package [28] in RStudio [29]. We included other explanatory variables in the analysis, such as the weeks following the treatments in the 8-week intervals, the number of active water drains within a buffer of 150 m from each BG-Trap, photoperiod or the number of tourists visiting the garden (descriptive tables summarizing the variables used in the analysis and the climatic conditions during the study period can be found in Tables S1 and S2 of the Supplementary Material).

We employed the Kruskal–Wallis tests to identify any significant differences in the number of mosquitoes captured during the sampling months followed by the Dunn test for pairwise comparisons (α = 0.05) since the counts of mosquitoes did not conform to a normal distribution. We also performed Wilcoxon–Mann–Whitney tests to assess differences in the average number of mosquitoes captured by sex (see Supplementary Material, Figures S1 and S2).

We developed Generalized Linear Mixed Models (GLMMs) with negative binomial distribution to investigate whether adult *Ae. albopictus* activity was influenced by changes in the variables above mentioned over the sampling period. The ID of each of the BG-Traps used for sampling was added as random effects in the models, as the conditions at each trap location were very different depending on the garden area and in terms of wind exposure, irrigation, humidity levels, or proximity to visitors’ pathways. We computed a full model including all explanatory variables and carried out step-wise backward deletion to choose the most adequate model, taking into account the variables’ collinearity. All analyses were performed using RStudio software version 4.2.0. The GLMMs were computed with the package *lme4* [30]. For model selection, we used the *MuMIn* package and the dredge() and model.sel() function, to which we added a function to combine the variables that did not show high collinearity (see Supplementary Material, “R function for collinearity”). Collinearity measurements were performed with the *car* package [31] and the vif() function. Finally, model validations were performed with the DHARMa package [32]. The calculation of water drains within 150 m of each BG-Trap was computed in QGIS v3.22.1 [33].

## 3. Results

**Figure 2:**
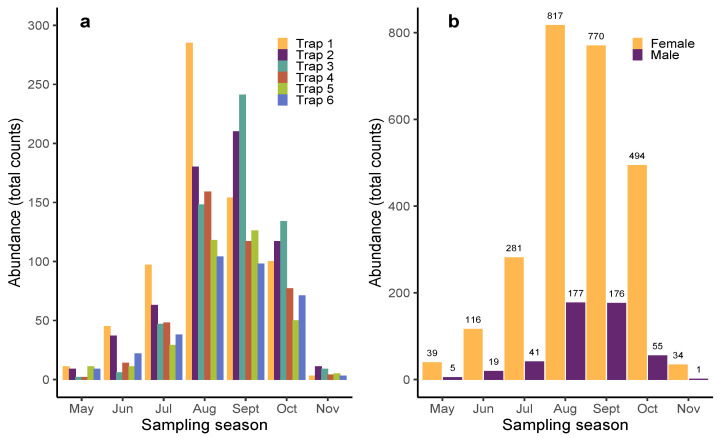
(**a**) Number of mosquitoes captured by trap and month. (**b**) Number of mosquitoes captured segregated by sex and month.

During the sampling period from May to November 2021, a total of 3025 individuals of the mosquito species *Aedes albopictus* were collected within the botanical garden. The amount of captured mosquitoes exhibited notable differences in terms of sex (refer to Supplementary Material, Figure S1), with 2551 females and 474 males recorded. Monthly fluctuations in the number of captured individuals are given in Figure 2, segregated by trap (Figure 2a) and by sex (Figure 2b). Statistical analysis revealed strong differences between the mosquito abundance at the beginning and end of the sampling season (May–June and November) compared to the months of peak abundance (August, September–October) (see Supplementary Material, Figure S2).

**Figure 3:**
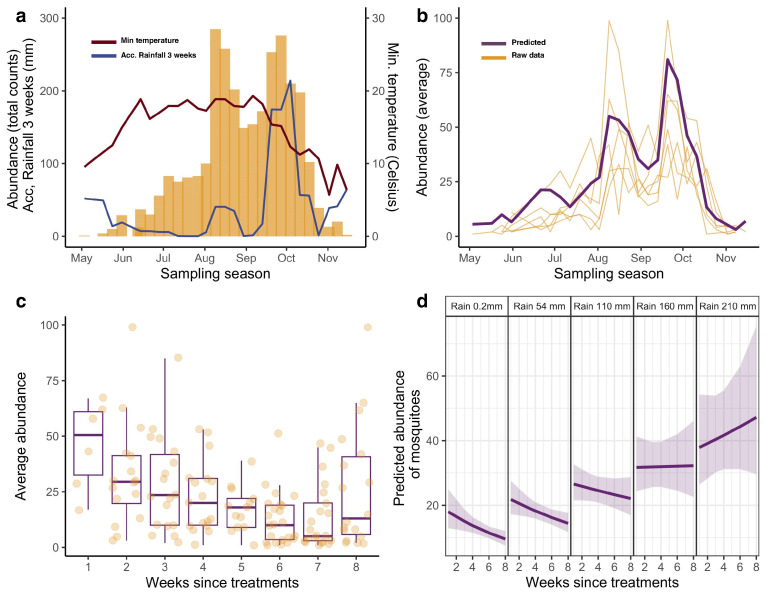
(**a**) Number of mosquitoes captured during the season (orange bars); red line indicate the minimum temperatures during the sampling season; blue line indicate the accumulated rainfall in the 3 weeks prior to each sampling week. (**b**) Number of mosquitoes predicted (in purple) with the GLMM model; in light orange, raw data of the number of mosquitoes captured by each trap. (**c**) Boxplot of average number of mosquitoes captured in the weeks following each treatment intervention. To assess the impact of abundance of treatment interventions, we aggregated count data on traps on a weekly basis, taking the intervention week as the reference week. (**d**) Interaction effect on predicted mosquito density in the weeks following treatments with increasing amounts of rainfall accumulated in the 3 weeks prior to sampling; at increasing amounts of accumulated rainfall in the 3 weeks prior to sampling, the detrimental effect of larvicide on the predicted number of mosquitoes subsequently captured is attenuated.

We also observed that adult mosquito captures followed a bimodal pattern during the sampling season, with two peaks of higher density, one at the onset of August and the other at the end of September (Figure 3a). We also observed that the gradual increase in temperatures at the beginning of the sampling weeks coincided with the increase in the number of adult mosquitoes captured and the overall U-inverted seasonal abundance pattern, while the second peak and other strong fluctuations in abundance coincided with high enough accumulated values of rainfall events that occurred over the season, particularly in September (Figure 3a; for detailed information, see Supplementary Material, Figure S3).

The best-fitting minimal model that explained the variations in the abundance of *Ae. albopictus* included meteorological variables such as the average of minimum temperatures and cumulative rainfall in the 3 weeks prior to mosquito captures (Table 1; Figure 3b), as well as the maximum relative humidity. In addition, other factors such as (i) the presence of drains with water in a 150 m buffer around each BG-Trap, (ii) the number of visitors at the botanical garden, and (iii) the larvicide treatments (with delayed effects) had an impact on the abundance of *Ae. albopictus* (Table 1). Our selected GLMM model (Table 1) accounted for a high proportion of the variance in our data; both fixed and random effects determined a conditional R2=0.803, and fixed effects alone, a marginal R2=0.793.

Importantly, we observed a detrimental (and delayed) effect on the number of *Ae. albopictus* adults captured in the traps in the weeks following larvicide treatment (Table 1). However, treatment efficacy in reducing mosquito numbers slowed down and decreased over time, with mosquito numbers increasing from the seventh week post-control onwards (Figure 3c).

**Figure 4:**
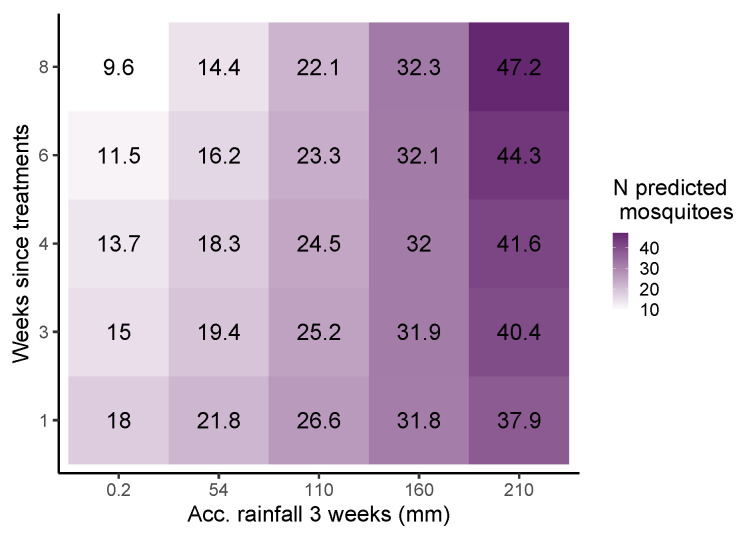
Predictions obtained from our model (Table 1), providing a visualization of how the interaction between the accumulated precipitation and the weeks since each treatment affects the number of captured mosquitoes. This figure shows in more detail the effect of accumulated rainfall on the control effectiveness extracted from Figure 3d.

We also observed a strong interaction in the potential efficacy of larvicidal treatments with previous cumulative rainfall values (Figure 3d). Higher values of accumulated rainfall predicted a shorter duration of treatment efficacy over subsequent weeks and a larger number of mosquitoes captured in the traps (Figure 4; for detailed information, see Supplementary Material, Figure S4). Other variables, such as the number of drains with water at the 150 m buffer from each BG-Trap, acting as potential developmental sites and potential sources of adults, also increased the number of mosquitoes. Finally, the presence of visitors at the botanical garden also showed a positive influence on the number of mosquitoes captured in the traps (Table 1). Visitors may promote a positive feedback to mosquito populations by providing blood meals to females, which then lay eggs in nearby water pools. Despite the presence of visitors in the botanical garden being strongly coupled to the seasonal temperature pattern (with a peak in August), the model shows that the positive effect on mosquito numbers is not only directly related to temperature.

## 4. Discussion

*Ae. albopictus* is a competent vector for dengue, Zika, and chikungunya viruses, known for its adaptability and invasive capacity at global scales [34,35]. The World Health Organization has long emphasized the management of mosquito populations as an essential strategy to combat the increasing global burden of mosquito-borne diseases in the absence of viable vaccines [36]. The effective management of mosquito populations requires integrative measures, including surveillance and control adaptive strategies across scales [37]. Mosquito surveillance demands understanding the ecological responses of mosquito populations to environmental change. This is particularly important at the local scale, where mosquito control occurs. Hence, anticipating local mosquito population responses is crucial to determining the appropriate timing and location of control measures to reduce mosquito populations and minimize pathogen transmission risks.

Building on the significance of ecological dynamics, climatic conditions are known to influence the transmission patterns and expansion of vector-borne diseases, directly impacting the behavior of pathogens, vectors, non-human hosts, and human populations. Climate change also has the potential to transform ecosystems, including urban environments, possibly enhancing or diminishing habitats for vectors and non-human hosts [38].

Our study revealed that *Ae. albopictus* exhibited a bimodal seasonal abundance pattern, characterized by two peaks, one occurring at the onset of August and the other towards the end of September. Our findings align with previous research [21,24,39] documenting the presence of these two abundance peaks and highlighting that the population of tiger mosquitoes remains significant even beyond the summer months. This pattern is anticipated to be exacerbated by climate change in Mediterranean regions [40]. However, while prior studies have noted this bimodal pattern, our analysis provides further insight into the underlying mechanisms. Temperature (i.e., average minimum temperatures) and rainfall (cumulative rainfall) over the preceding 3 weeks to mosquito sampling emerged as the primary determinants of the mosquito seasonal abundance pattern. By controlling for these two variables, our model predicts that the overall U-inverted seasonal pattern of *Ae. albopictus* abundance is primarily driven by the preceding temperatures, while the peaks (strong abundance fluctuations) are predominantly influenced by preceding rainfall events. Temperature shows the largest regression coefficient and weight deviance compared to other significant factors, consistent with previous observations [21,41]. The weight deviances for the rest of the factors were of similar magnitude, suggesting that other than temperature, the local seasonality of tiger mosquitoes is shaped by a diverse array of determinants, including both environmental and human-related variables, each contributing similarly to mosquito abundance. For example, the peaks observed in August and September coincided with a heightened influx of visitors at the botanical garden, particularly notable in August. We hypothesize that this increased tourist activity may have augmented host availability for blood-feeding female mosquitoes, potentially contributing to the observed abundance peaks during these periods.

Given the poikilothermic nature of insects, mosquitoes are highly susceptible to temperature fluctuations, directly impacting their body temperature [14]. Previous investigations on *Ae. albopictus* underscore the pivotal role of temperature in its population ecology [42,43], with temperatures ranging from 25 °C to 30 °C identified as optimal for development [42]. In laboratory conditions, Alto and Juliano [41] showed that elevated temperatures can adversely affect adult mosquito presence when coupled with drying conditions. The observed reduction in mosquito captures during August might be attributed to high temperatures and dry conditions, negatively impacting host-seeking behavior (minimizing capture rates) and reducing breeding site proliferation through water evaporation. In this context, although plants were watered regularly by the botanical garden workers, they followed good practices to avoid the proliferation of immature developmental sites (e.g., emptying containers with accumulated water and monitoring water drains that might be clogged). Therefore, rain events could play a crucial role, promoting the emergence of new sites for breeding and the reactivation of previously dormant ones. With some time delay, rain events trigger the hatching of a considerable number of eggs laid by females, contributing to the observed abundance peaks in adults. The positive correlation between preceding rainfall and mosquito presence aligns with findings also in other studies [44,45,46]. Nonetheless, some authors noted a lack of correlation between abundance and rainfall [47], or in some cases, even a negative correlation [21]. The latter was attributed to the presence of human-mediated water supplies, which mask the impact of rainfall on the variation of mosquito abundance, while the lack of correlation found between rainfall and abundances in Luciano et al. [47] may be due to the fact that time lags were not considered. Prolonged and heavy rains may also have a flushing effect on larval breeding sites, depleting subsequent adult populations [21,48,49].

We were also able to analyze the impact of the larvicide control program on the abundance of mosquitoes. We observed that after applying larvicide (i.e., Vectomax^®^ FG), the number of captured mosquitoes in the following weeks was strongly reduced. The most prominent decline in the adult mosquito population was evident approximately from the fourth week after the treatment. However, the adverse impact on mosquito populations decreased by the seventh week post-treatment. We observed a significant interaction between the accumulated rainfall and the efficacy of Vectomax^®^ FG in the weeks post-treatment. The analysis indicated that when there was limited cumulative rainfall in the three weeks before sampling, reflecting a scarce rainfall history, the treatment significantly reduced adult mosquito abundance in the following weeks. In contrast, intense rainfall historical events leading to an increase in cumulative rainfall reduced the effectiveness of the larvicide. Several factors could contribute to this depletion in treatment effect. First, heavy rain events may have diluted the treatment, washing it away. Second, the rain events could have triggered the emergence of new, previously undetected, temporary breeding sites near or within the botanical garden (e.g., plastic buckets and tree holes), resulting in increased larval densities and higher mosquito capture rates in the following weeks. Finally, runoff caused by heavy rainfall may have washed large amounts of organic matter into the drains, competing with the larvicide for ingestion by the larvae.

The impact of rainfall on the population dynamics of *Ae. albopictus* is underscored by various studies [50,51]. Rainfall can significantly affect larval control activities by diminishing the efficacy and persistence of products, stimulating egg hatching, and dispersing larvae and pupae. Thus, when devising larvicide treatments with extended intervals, it is imperative to account for environmental factors that influence their effectiveness and durability. Our research emphasizes the role of rainfall and natural product degradation, often accelerated by larval consumption, in shaping the efficacy of treatments. For instance, Ravasi et al. [25] demonstrated that Vectomax^®^ reached peak effectiveness against *Ae. albopictus* between weeks 5 and 10, resulting in a 60% reduction in adult emergence from catch basins. Similarly, Guidi et al. [52] observed a significant decrease in immature mosquitoes for at least 10 weeks following a single application of 10g of *Bti* per catch basin [52]. In our study, larvicide treatments led to a 13% reduction in adult *Ae. albopictus* captures per week post-treatment. Assuming an optimal 8-week period to maximize a reduction in the number of mosquitoes (as observed in traps), and discounting immigration and complex larvicide–rainfall interactions, we could potentially reduce the population to 33%. However, the presence of additional untreated breeding sites due to rainfall, coupled with inadequate water drainage systems, may have partially obscured the impact of treatments. Therefore, effective control strategies should consider preceding rainfall patterns and incorporate monthly weather forecasts to prevent water flushing in treated breeding sites and the emergence of new sites suitable for development. In addition, the targeted removal of highly productive breeding sites should be complemented by treatments in surrounding areas, as *Ae. albopictus* females tend to lay eggs in less suitable containers when productive sites are unavailable [53].

This study demonstrated certain limitations. Firstly, a basic assumption in mosquito seasonal studies (and maybe interpretable as a limitation) is that mosquito traps only capture a small and constant proportion of adults from real populations [24,54] but can properly reflect the abundance patterns in the area. Secondly, obtaining detailed climatic data at the specific locations of each BG-Trap in the garden would have enabled a more precise analysis of the variables influencing abundance in each environment. The botanical garden features areas of great diversity, with various types of vegetation (ranging from tropical regions to arid zones), and the traps were strategically distributed across these diverse environments. Each trap was influenced by different degrees of humidity, wind exposure, and irrigation. To account for the variability in capture data from each trap, we incorporated it as a random effect in the analysis, which included both local environmental variation and the possible malfunctioning of specific traps, leading to potential capture biases. Nevertheless, future studies should consider obtaining climate data at a finer scale, allowing for a more detailed analysis of the variables affecting the population dynamics of these species. Finally, a shortcoming that needs to be addressed in future control optimization studies is the monitoring of many other breeding sites within and around the botanical garden, not included in our monitoring. The emergence of new breeding sites is quite dynamic over the season, and *Ae. albopictus* females are good at exploiting them as discussed above.

Our research has highlighted the importance of studying vector ecology at a local scale to enhance the efficacy of control programs and address the escalating burden of vector-borne diseases. By recognizing the potential for climate change to intensify or extend transmission seasons through warmer temperatures and more intense but erratic rainfall events, we understand that future extreme weather events can heavily influence mosquito abundance and disease risk. Therefore, it is essential to incorporate the effects of these variables into our mechanistic understanding of the ecological patterns and processes of mosquito populations. This approach will allow us to design targeted and sustainable control programs that are not only adapted to local mosquito populations but are also robust to the challenges imposed by a changing climate. Consequently, this will help us to more effectively reduce the incidence and prevalence of vector-borne diseases.

## Figures and Tables

**Table 1:** Model estimates and Deviance Analysis (Anova Type III) of the best fitting Generalized Linear Mixed model (GLMM) with negative binomial distribution explaining *Aedes albopictus* abundance in the botanical garden.

Response Variable	Explanatory Variables	Coefficient (±SE)	(95% CI)	IRR (95% CI)	Wald Test	$\chi^{2}$	df	*p*-Value
Abundance of	Intercept	2.78 ± 0.06	(2.66, 2.91)	16.25 (14.33, 18.43)	43.392	1882.87	1	<0.0001
mosquitoes	MaxRH	0.23 ± 0.05	(0.13, 0.32)	1.25 (1.14, 1.38)	4.74	22.54	1	<0.0001
	MinTemp—21	0.58 ± 0.06	(0.47, 0.70)	1.80 (1.60, 2.01)	10.04	100.87	1	<0.0001
	N of Visitors	0.23 ± 0.05	(0.13, 0.34)	1.26 (1.14, 1.41)	4.43	19.62	1	<0.0001
	Number of water drains with water	0.27 ± 0.06	(0.15, 0.39)	1.31 (1.16, 1.48)	4.37	19.13	1	<0.0001
	Weeks since treatments * Acc. Rainfall 3w	0.07 ± 0.03	(0.003, 0.14)	1.07 (1.00, 1.15)	2.05	4.23	1	0.038
	Acc. Rainfall 3w	0.35 ± 0.05	(0.25, 0.45)	1.42 (1.28, 1.57)	6.90	47.67	1	<0.0001
	Weeks since treatments	−0.13 ± 0.05	(−0.24, −0.029)	0.87 (0.78, 0.97)	−2.49	6.23	1	0.012

The symbol * indicates interaction between variables.

## Data Availability

The original data presented in the study is available in the following Github repository: https://github.com/lblancozgz/Human-Environment-Interactions-shape-Mosquito-Seasonal-Population-Dynamics.

## Supplementary Materials

The following supporting information can be downloaded at: https://www.mdpi.com/article/10.3390/insects15070527/s1, Table S1: Summary of the variables calculated for the GLMM analysis. Table S2. Summary of the climatic conditions during the study period in the botanical garden. Figure S1: Significant differences in the average number of mosquitoes captured by sex after performing Wilcoxon–Mann–Whitney test. Figure S2: Significant differences in the number of mosquitoes captured by month after performing Kruskall–Wallis and post-hoc Dunn test. R function for collinearity. Figure S3: Influence of temperature and rainfall on predicted mosquito abundance during the sampling season. Figure S4: Predictions based on varying levels of accumulated rainfall and the comparison of predicted mosquito captures in two different weeks post-treatment (Week 1 and Week 6).
