# Supplementary material for: Human–Environment Interactions Shape Mosquito Seasonal Population Dynamics
Source: Insects. 2024 Jul 12;15(7):527. doi: 10.3390/insects15070527 (PMC11276872; doi:10.3390/insects15070527)
Supplement: Supplementary file 1 [file insects-15-00527-s001.zip › insects-3038225-supplementary.pdf]

Table S1. Summary of the variables calculated for the GLMM analysis

Variable name	Description
Min temperature	Minimum temperature of the sampling week
Mean temperature	Mean temperature of the sampling week
Max temperature	Max temperature of the sampling week
MinTemp - 7	Average minimum temperature of the 7 days previous to sampling
MeanTemp - 7	Average mean temperature of the 7 days previous to sampling
MaxTemp - 7	Average max temperature of the 7 days previous to sampling
MinTemp - 14	Average minimum temperature of the 14 days previous to sampling
MeanTemp - 14	Average mean temperature of the 14 days previous to sampling
MaxTemp - 14	Average max temperature of the 14 days previous to sampling
MinTemp - 21	Average minimum temperature of the 21 days previous to sampling
MeanTemp - 21	Average mean temperature of the 21 days previous to sampling
MaxTemp - 21	Average max temperature of the 21 days previous to sampling
Min RH	Minimum relative humidity of the sampling week
Mean RH	Mean relative humidity of the sampling week
Max RH	Max relative humidity of the sampling week
MinRH - 7	Average minimum relative humidity of the 7 days previous to sampling
MeanRH - 7	Average mean relative humidity of the 7 days previous to sampling
MaxRH - 7	Average max relative humidity of the 7 days previous to sampling
MinRH - 14	Average minimum relative humidity of the 14 days previous to sampling
MeanRH - 14	Average mean relative humidity of the 14 days previous to sampling
MaxRH - 14	Average max relative humidity of the 14 days previous to sampling
MinRH - 21	Average minimum relative humidity of the 21 days previous to sampling
MeanRH - 21	Average mean relative humidity of the 21 days previous to sampling
MaxRH - 21	Average max relative humidity of the 21 days previous to sampling
GDD	Growing Degree Days
GDD - 7	GDD calculated for the 7-day lag previous to sampling
GDD - 14	GDD calculated for the 14-day lag previous to sampling
GDD - 21	GDD calculated for the 21-day lag previous to sampling
Photoperiod	(hrs light/day)
Rainfall	Precipitation during the sampling week
Rainfall - 7	Sum of precipitation during the 7 days previous to sampling
Rainfall - 14	Sum of precipitation during the 14 days previous to sampling
Rainfall - 21	Sum of precipitation during the 21 days previous to sampling
Visitors	Number of Visitors during the sampling week
Visitors - 7	Sum of Visitors during the 7 days previous to sampling
Visitors - 14	Sum of Visitors during the 14 days previous to sampling
Visitors - 21	Sum of Visitors during the 21 days previous to sampling
WD150	Number of water drains at 150 meters
WDL	Water drains with presence of larvae
WDW	Water drains with presence of water
WST	Weeks since treatments

Table S2. Summary of the climatic conditions during the study period in the botanical garden.

**Month**	**MeanTemp (°C)**	**MinTemp (°C)**	**MaxTemp (°C)**	**Total Rainfall (mm)**	**MeanRH (%)**	**MinRH (%)**	**MaxRH (%)**
May	15.94 ± 1.81	10.84 ± 2.47	20.35 ± 2.10	48.5	80.70 ± 5.91	61.06 ± 8.79	94.83 ± 3.04
June	22.17 ± 1.61	16.63 ± 1.93	27.1 ± 1.91	7.2	74.7 ± 7.02	54.2 ± 9.49	91.23 ± 5.99
July	24.04 ± 1.12	18.10 ± 1.21	28.55 ± 1.34	5.8	72.74 ± 6.43	54.51 ± 7.15	89.35 ± 4.67
August	23.33 ± 1.24	18.09 ± 1.66	28.32 ± 1.40	35.7	74.58 ± 7.42	54.12 ± 9.39	91.96 ± 4.65
September	22.1 ± 2.11	17.51 ± 2.39	27.02 ± 1.81	210.1	80.76 ± 4.53	60.26 ± 8.11	95.16 ± 2.54
October	17.06 ± 2.04	12.11 ± 2.61	22.29 ± 1.28	54.3	79.48 ± 6.96	58.22 ± 12.08	95.16 ± 2.74
November	11.13 ± 2.61	6.63 ± 3.58	16.27 ± 2.20	103	77.43 ± 10.73	54.86 ± 14.50	93 ± 7.51

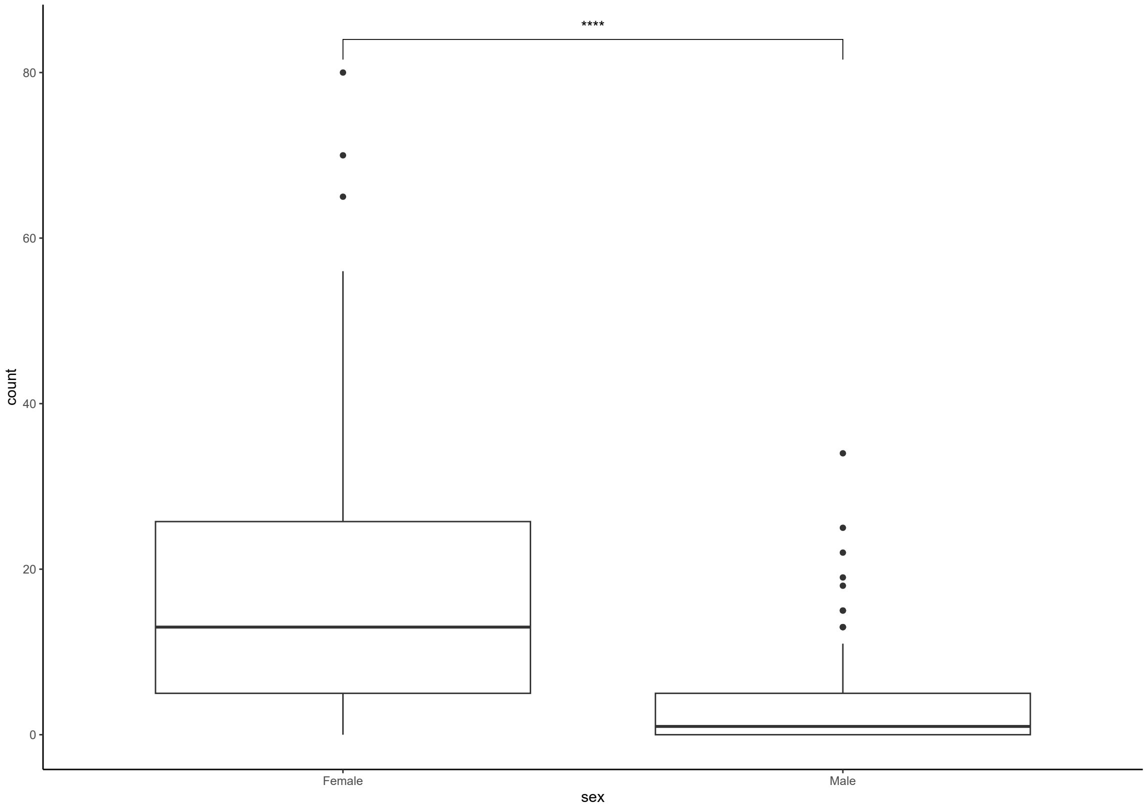

Figure S1. Significant differences in the average number of mosquitoes captured by sex after performing Wilcoxon-Mann Whitney test.

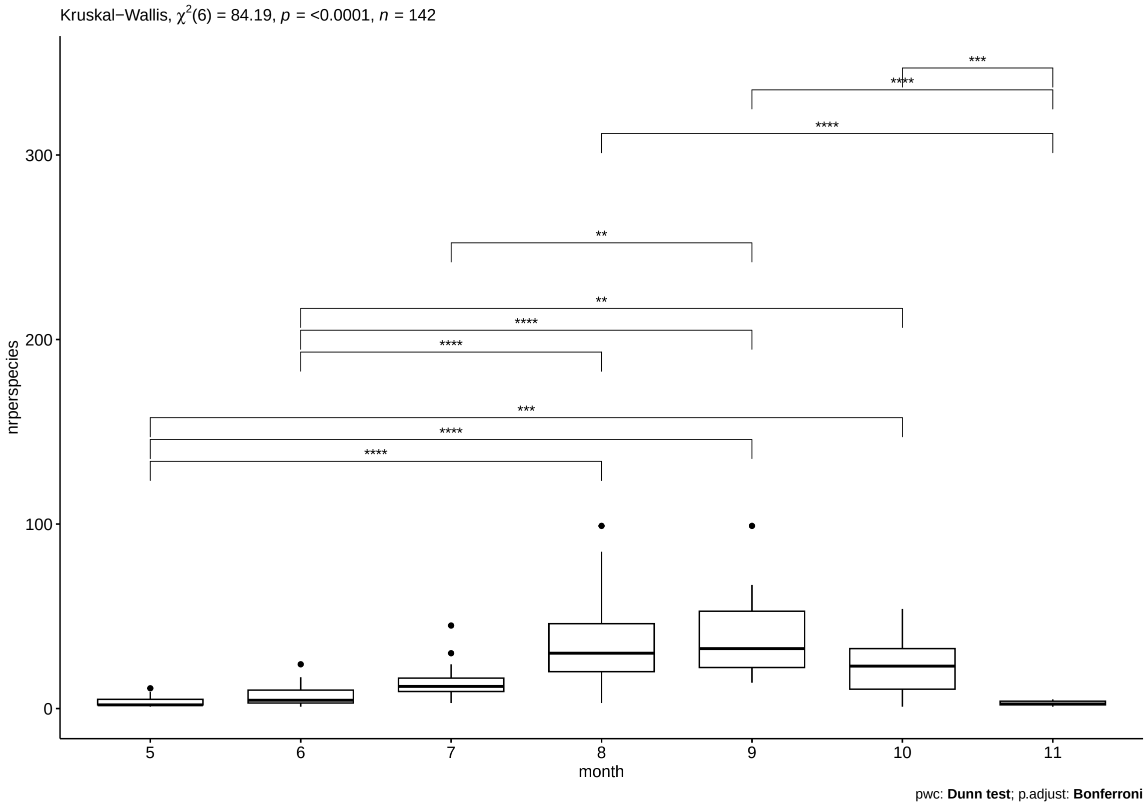

Figure S2. Significant differences in the number of mosquitoes captured by month after performing Kruskall-Wallis and *post-hoc* Dunn-test.

R function for collinearity

```r
max.r <- function(x){
  corm <- cov2cor(vcov(x))
  corm <- as.matrix(corm)
  if (length(corm)==1){
    corm <- 0
    max(abs(corm))
  } else if (length(corm)==4){
    cormf <- corm[2:nrow(corm),2:ncol(corm)]
    cormf <- 0
    max(abs(cormf))
  } else {
    cormf <- corm[2:nrow(corm),2:ncol(corm)]
    diag(cormf) <- 0
    max(abs(cormf))
  }
}
```

Link = Github source

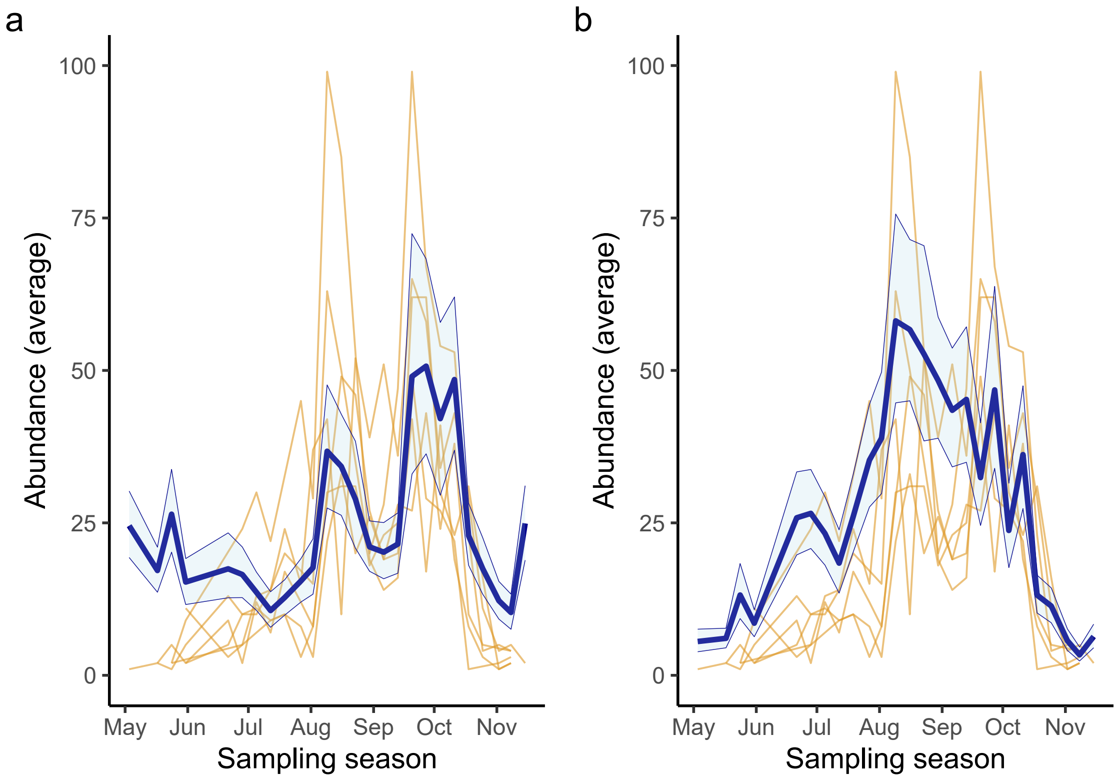

Figure S3. Influence of temperature and rainfall on predicted mosquito abundance during the sampling season. The orange lines show the number of mosquitoes captured per trap over time, and is the same for both panels. The blue line represents the predicted abundance from the model (see Table 1). In **a)**, the prediction is made setting a constant temperature value (mean of the minimum temperature from the 3 weeks before each sampling week), while the other covariables fluctuate. In **b)**, the prediction is made setting a constant accumulated precipitation value, while the other covariables fluctuate.

In both figures, the first peak in mosquito abundance occurs in early August. However, when rainfall is kept constant (Fig. S3b), the bimodal pattern (typically showing a second peak in September-October) disappears. This suggests that variations in rainfall might be crucial for explaining the second peak, indicating a potential link between rainfall events and the observed bimodal pattern in mosquito abundance.

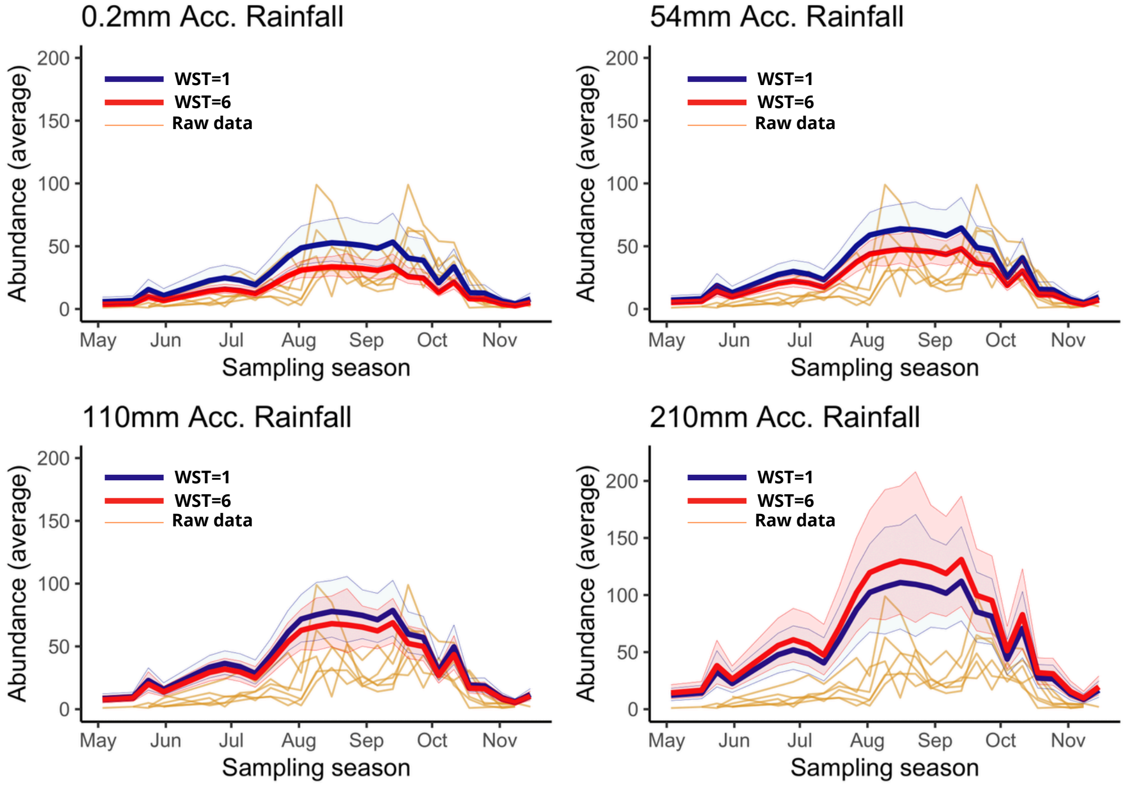

Figure S4. Predictions based on varying levels of accumulated rainfall and the comparison of predicted mosquito captures in two different weeks post-treatment (Week 1 and Week 6). The orange lines represent the actual number of mosquitoes captured per trap over time (raw data) and is the same in all the panels. The blue line indicates the predicted abundances in the first week after larvicide applications, setting constant fixed accumulated rainfall values while other model covariables fluctuate (model in Table 1). The red line indicates the predicted abundances in the sixth week after larvicide applications, also setting constant fixed accumulated rainfall values while other model covariables fluctuate.

When accumulated precipitation values are very low (e.g., 0.2 mm), the predicted number of mosquitoes captured is notably lower compared to higher precipitation values (e.g., 210 mm). Therefore, with the rise in cumulative rainfall values observed during the study period, we observed a corresponding increase in the predicted mosquito abundance. At 210 mm of rainfall, the predicted number of mosquitoes in Week 6 (red line) is already very high compared to the first week after treatment (blue line), as the rains could have washed away the larvicide or created new breeding sites.
